# Clinical characteristics, treatment patterns, and seizure control among children with focal and generalized epilepsy at a tertiary hospital in Tanzania: A cross-sectional secondary analysis

**DOI:** 10.1371/journal.pone.0344724

**Published:** 2026-04-02

**Authors:** Erneus Ernest

**Affiliations:** Department of Paediatrics and Child Health, Mbeya College of Health and Allied Sciences, University of Dar es Salaam, Mbeya, Tanzania; University of Diyala College of Medicine, IRAQ

## Abstract

This study aimed to describe the clinical characteristics and treatment patterns of children with focal and generalized epilepsy and to identify factors associated with seizure control at a tertiary hospital in Tanzania. We conducted a hospital-based cross-sectional secondary analysis of 427 children and adolescents (aged 0–17 years) with a clinical diagnosis of epilepsy whose caregivers were enrolled in a primary study between June and October 2023 at the pediatric neurology clinic of a national referral hospital in Dar es Salaam. Primary outcomes included epilepsy type distribution, treatment patterns (monotherapy vs. polytherapy and specific antiseizure medications), and level of seizure control. The secondary outcome was the identification of factors associated with seizure control, analyzed using ordinal logistic regression and reported as adjusted odds ratios (aORs). Focal epilepsy was the predominant type (52.5%). Most children (65.8%) had infantile seizure onset (<1 year) and were receiving monotherapy (74.9%). Treatment patterns were consistent with guideline recommendations: carbamazepine was prescribed to 94.6% of children with focal epilepsy, while sodium valproate was used in 96.4% of those with generalized epilepsy (both p < 0.001). Seizure freedom in the preceding month was higher among children with focal epilepsy (73.7%) compared with generalized epilepsy (59.5%, p = 0.003). In multivariable analysis, generalized epilepsy was independently associated with worse seizure control (aOR=1.93, 95% CI: 1.28–2.92, p = 0.002). Focal epilepsy was the most common type in this tertiary referral cohort, and treatment largely aligned with international guidelines. Children with generalized epilepsy were more likely to have poorer seizure control. Further prospective studies incorporating detailed syndromic and etiologic classification are needed to better define predictors of seizure control and inform service planning.

## Introduction

Over the decades, epilepsy has remained one of the most common neurological conditions affecting about 45 million people globally [1]. There remains a disproportionate distribution of the epilepsy burden, with low- and middle-income countries (LMICs) occupying the lion’s share [2].

Epilepsy remains more common in children and young adults [3], with the prevalence of childhood epilepsy ranging from 3.6–44/1000 in developing countries, while a lower prevalence (3.2–5.5/1000) is reported in developed countries [3].

The management of epilepsy is complex, with pharmacotherapy using antiseizure medication (ASM) being the mainstay. Other treatment modalities are indicated in patients refractory to pharmacotherapy and include immunotherapy, hormonal therapy, ketogenic diet, neurostimulation, and surgery [1].

Nearly half of the patients become seizure-free with the first ASM that is prescribed [4]. The selection of initial medication is complex and relies on several factors, including its effectiveness for a particular seizure type or syndrome, comorbidities, and potential drug interactions [5].

Narrow-spectrum antiseizure drugs like carbamazepine are preferred for focal epilepsies, as most medications show comparable efficacy against focal seizures [1]. In contrast, for generalized epilepsies, broad-spectrum agents like sodium valproate are recommended due to demonstrated superior efficacy over many alternatives [6].

Evidence shows that up to 70% of people living with epilepsy can be seizure-free when appropriate diagnosis and treatment are provided. However, treatment gaps are noted across different geographical locations, with LMICs having a treatment gap exceeding 75% [2].

The treatment gaps are attributable to multiple factors, including a scarcity of neurologists in LMICs, limited availability and high costs of ASMs, societal and cultural influences leading to the stigmatization of epilepsy patients, and insufficient awareness and acceptance of treatment options. Conversely, deaths due to epilepsy are higher in LMICs [2]. Compounding this problem, even for children who access specialist care, data to guide service improvement are scarce.

In Tanzania, key questions remain regarding the local distribution of epilepsy types, age of onset, associated comorbidities, and the level of seizure control achieved. Characterizing this profile is essential to benchmarking the quality of care, identifying local risk factors for poor outcomes, and informing targeted interventions and resource planning.

Therefore, this study aimed to (1) describe the clinical characteristics and treatment patterns of children with focal and generalized epilepsy, and (2) identify factors associated with seizure control at a tertiary hospital in Tanzania.

## Materials and methods

### Study design and setting

We conducted a hospital-based, cross-sectional secondary analysis of data. The data were derived from a primary study conducted between June and October 2023, which investigated mental health outcomes among caregivers of children and adolescents with epilepsy attending the pediatric neurology clinic at Muhimbili National Hospital (MNH) in Dar es Salaam, Tanzania (S1 File).

Although the primary focus was on caregivers, the study protocol included comprehensive clinical data collection for each child to characterize the patient population. These data included sociodemographic characteristics, epilepsy type, age at seizure onset, current antiseizure medications, comorbidities, and recent seizure frequency.

This available dataset provided a unique opportunity to address a distinct and relevant research question: to describe the clinical characteristics and treatment patterns of the children themselves.

MNH is a national referral hospital and university teaching hospital located in Dar es Salaam, Tanzania. The pediatric neurology clinic operates within the pediatric department and is conducted twice a week. The clinic is staffed by two consultant pediatric neurologists, supported by pediatricians.

### Study population, sample size, and sampling method

The study population for this secondary analysis comprised all children and adolescents (aged 0–17 years) with a clinical diagnosis of epilepsy whose caregivers were enrolled in the primary study at the MNH pediatric neurology clinic.

The primary study enrolled caregivers of children with epilepsy (CWE) who met the following criteria: 1) being the primary caregiver of a child (0–17 years) with a clinical diagnosis of epilepsy diagnosed by a pediatric neurologist, and 2) having provided care for the child for at least six months before the commencement of the study. Caregivers were enrolled via consecutive enrollment between June and October 2023.

Consequently, for this secondary analysis, the child cohort was defined indirectly through their eligible caregiver. Each enrolled caregiver provided detailed clinical and demographic information for the one associated child with epilepsy. Thus, the 427 children included in this analysis represent those whose caregivers met the eligibility criteria and consented to participate, rather than all children with epilepsy seen at the MNH pediatric neurology clinic during the study period.

### Data collection

Data were collected during the primary study period from June to October 2023 through structured interviews and hospital medical records. Structured interviews with enrolled caregivers were conducted by the primary investigator (a pediatrician) and a trained research assistant (a qualified medical doctor) to obtain sociodemographic information and caregiver-reported clinical history, including the child’s age at seizure onset, history of seizure-related injuries, and frequency of hospital admission. Seizure control was assessed based on caregiver-reported seizure frequency over the preceding one month, as documented during the clinic visit and verified against seizure diaries when available. This time frame was selected to balance clinical relevance with caregiver recall accuracy in a cross-sectional clinic-based setting. As part of routine clinic care, caregivers are advised to record seizure occurrences; when seizure diaries were available at the clinic visit, reported seizure frequency was verified against the diary. For caregivers without diaries, seizure frequency was obtained through caregiver recall during the interview. In parallel, the primary investigator abstracted key clinical data from the hospital medical records, including the neurologist-documented seizure type and current ASM. All abstracted clinical information was cross-checked with the caregiver during the interview to enhance data accuracy. Epilepsy type was classified according to the ILAE 2017 framework based on clinical diagnoses made by a pediatric neurologist during routine care. Classification into focal or generalized epilepsy was primarily based on detailed clinical semiology documented by a pediatric neurologist in the medical records, including seizure onset features and evolution. Electroencephalography and neuroimaging were used to support the clinical diagnosis where available; however, these investigations were not systematically collected as variables for the primary study and were therefore unavailable for inclusion in this secondary analysis.

The presence of neurodevelopmental comorbidities was determined through a combination of medical record review and direct clinical assessment of the child during the clinic visit. For analysis, neurodevelopmental comorbidity was treated as a binary variable, defined as the presence of at least one neurodevelopmental condition (including cerebral palsy, intellectual disability, autism spectrum disorder, or attention-deficit/hyperactivity disorder) versus no neurodevelopmental condition, as individual conditions were relatively infrequent and severity was not systematically captured in the primary study, making separate analyses of specific conditions statistically underpowered.

This study follows the Checklist for Reporting of Survey Studies (CROSS) guidelines; the completed checklist is provided as an S2 File.

### Ethics statement

The original data collection was conducted in accordance with the Declaration of Helsinki and was approved by the Institutional Review Board of Muhimbili University of Health and Allied Sciences (MUHAS-REC-05-2023-1679). Written informed consent was obtained from caregivers at the time of the primary study. For this secondary analysis, the dataset was accessed in anonymized form under the original ethical approval on or around 15 November 2025. The author did not have access to information that could directly identify individual participants during or after data analysis.

### Data analysis

Data were analyzed using STATA version 18. Categorical variables were summarized as frequencies and proportions (percentages), and continuous variables as means with standard deviations (SD).

Clinical and demographic characteristics were compared between children with focal and generalized epilepsy using the chi-square test for categorical variables (Table 1). For this comparative analysis, eight participants were excluded: five with epilepsy of unknown onset and three with combined focal and generalized epilepsy, resulting in an analytical sample of 419 participants (224 focal, 195 generalized).

**Table 1:** Sociodemographic and clinical characteristics of children with epilepsy by seizure type.

VARIABLE	SUBTYPE	Focal (N = 224)	Generalized (N = 195)	P-value
**Age (years)**	Under 5 years	89 (39.7%)	75 (38.5%)	
	5-11 years	111 (49.6%)	97 (49.7%)	
	12 + years (Adolescents)	24 (10.7%)	23 (11.8%)	
				0.927
**Sex**	Male	123 (54.9%)	107 (54.9%)	
	Female	101 (45.1%)	88 (45.1%)	
				0.994
**Education level**	Not formal education	106 (47.3%)	87 (44.6%)	
	Any formal education	118 (52.7%)	108 (55.4%)	
				0.579
**Age at seizure onset**	Infantile onset (< 1 year)	157 (70.1%)	118 (60.5%)	
	Childhood onset (1–11 years)	64 (28.6%)	75 (38.5%)	
	Adolescent onset (≥ 12 years)	3 (1.3%)	2 (1.0%)	
				0.099
**Neurodevelopmental comorbidity**	No neurodevelopmental comorbidity	163 (72.8%)	128 (65.6%)	
	≥ 1 neurodevelopmental comorbidity	61 (27.2%)	67 (34.4%)	
				0.114
**Seizures (past one month)**	Seizure free (No episode)	165 (73.7%)	116 (59.5%)	
	Well controlled (1–2 episodes)	48 (21.4%)	56 (28.7%)	
	Poorly controlled (≥ 3 episodes)	11 (4.9%)	23 (11.8%)	
				**0.003**
**Epilepsy related injuries**	No injury	203 (90.6%)	174 (89.2%)	
	Injury	21 (9.4%)	21 (10.8%)	
				0.635
**Number of hospital admissions**	No admission	93 (41.5%)	76 (39.0%)	
	1-2 admissions	109 (48.7%)	95 (48.7%)	
	≥ 3 admissions	22 (9.8%)	24 (12.3%)	
				0.686

***Footnotes:*** *This comparative analysis excluded eight children: five with epilepsy of unknown onset and three with combined focal and generalized epilepsy, resulting in an analytical sample of 419 participants (224 focal, 195 generalized).*

Treatment patterns were similarly compared by epilepsy type (Table 2). The use of monotherapy versus polytherapy and the specific antiseizure medications prescribed were analyzed using the chi-square test. For the analysis of current medication use, two additional participants who were not on any medication were excluded, resulting in a denominator of 417.

**Table 2:** Treatment patterns of children with epilepsy by epilepsy type.

PART A: TREATMENT APPROACH				
Treatment Pattern	Overall (N = 417)	Focal (N = 224)	Generalized (N = 193)	P-value
**Monotherapy**	314 (75.3%)	172 (76.8%)	142 (73.6%)	
**Polytherapy (≥2 drugs)**	103 (24.7%)	52 (23.2%)	51 (26.4%)	0.448
**PART B: SPECIFIC MEDICATION USE**				
**Valproate**	221 (53.0%)	35 (15.6%)	186 (96.4%)	**<0.001**
**Carbamazepine**	244 (58.5%)	212 (94.6%)	32 (16.6%)	**<0.001**
**Phenobarbitone**	28 (6.7%)	19 (8.5%)	9 (4.7%)	0.120
**Levetiracetam**	8 (1.9%)	6 (2.7%)	2 (1.0%)	0.223
**Clonazepam**	37 (8.9%)	15 (6.7%)	22 (11.4%)	0.092
**Other**	3 (0.7%)	3 (1.3%)	0 (0.0%)	0.107

***Footnotes:*** *The analytical sample for Table 2 is children with a classified epilepsy type (focal or generalized) who were receiving antiseizure medication (N = 417). This excludes: 5 children with epilepsy of unknown onset, 3 with combined focal and generalized epilepsy, and 2 children not on any medication. Percentages represent the proportion within each epilepsy type group prescribed a specific medication. Columns do not sum to 100% because patients on combination therapy are counted in each applicable medication row.*

To identify factors associated with seizure control, we performed an ordinal logistic regression. The outcome variable was seizure frequency in the past month, categorized into three ordered levels: seizure-free (no episodes), well-controlled (1–2 episodes), and poorly controlled (≥3 episodes). This approach was chosen to model the natural gradient of seizure severity and to preserve information that would be lost by dichotomizing the outcome. Variables for inclusion in the multivariable model were pre-selected based on clinical relevance and existing literature. Age at seizure onset was included as the primary age-related variable; current age was not included due to collinearity with age at onset. Sex was not included in the model as there is limited evidence to support an independent association between sex and seizure control in pediatric epilepsy outside specific syndromic contexts.

The proportional odds assumption was assessed by comparing the ordinal logistic regression model with a multinomial logistic regression model and was supported for all variables included.

In the final model, variables with an adjusted odds ratio (aOR) and a p-value <0.05 were considered independently associated with worse seizure control (Table 3).

**Table 3:** Factors associated with Worse Seizure Control (Ordinal Logistic Regression).

	Univariate analysis		Multivariate analysis	
**VARIABLE**	**cOR (95% CI)**	**P-value**	**aOR (95% CI)**	**P-value**
**Epilepsy type**				
Focal	1	Ref	1	
Generalized	1.96 (1.30-2.94)	0.001	1.93 (1.28-2.92)	**0.002**
**Age at onset**				
Infantile onset (< 1 year)	1	Ref	1	
Childhood onset (1–11 years)	1.15 (0.75-1.76)	0.519	1.11 (0.71-1.73)	0.658
Adolescent onset (≥ 12 years)	1.67 (0.27-10.21)	0.574	1.83 (0.28-11.80)	0.524
**Treatment Pattern**				
Monotherapy	1	Ref	1	
Polytherapy	1.19 (0.75-1.90)	0.447	1.18 (0.74-1.89)	0.490
**Neurodevelopmental comorbidities**				
No neurodevelopmental comorbidity	1	Ref	1	
≥ 1 neurodevelopmental comorbidity	1.10 (0.71-1.71)	0.647	1.06 (0.68-1.68)	0.789

***Footnotes:*** *The outcome variable was seizure control, an ordinal measure with three levels: 0 = seizure-free (no episodes), 1 = well-controlled (1–2 episodes), and 2 = poorly controlled (≥3 episodes). The proportional odds assumption was met. cOR = crude odds ratio; aOR = adjusted odds ratio; CI = confidence interval.*

## Results

Of the 427 children with epilepsy, the mean age was 6.2 ± 3.7 years, with nearly half (49.4%) aged 5–11 years. Slightly more than half were male (55.0%) and were diagnosed with focal epilepsy (52.5%). Infantile seizure onset (<1 year) was more common (65.8%). Neurodevelopmental comorbidities were present in 30.4% of the cohort. The full sociodemographic and clinical characteristics of the study population are detailed in Table 4.

**Table 4:** Sociodemographic and clinical characteristics of children with epilepsy (N = 427).

VARIABLE	SUBTYPE	FREQUENCY	PERCENT %
**Age (years)**	Under 5 years	168	39.4%
	5-11 years	211	49.4%
	12 + years (Adolescents)	48	11.2%
	Mean (± SD) 6.2 ± 3.7 years		
**Sex**	Male	235	55.0
	Female	192	45.0
**Education level**	Not formal education	198	46.4
	Any formal education	229	53.6
**Epilepsy type**	Focal	224	52.5
	Generalized	195	45.7
	Combined generalized and focal epilepsy	3	0.7
	Unknown	5	1.1
**Age at seizure onset**	Infantile onset (< 1 year)	281	65.8
	Childhood onset (1–11 years)	141	33.0
	Adolescent onset (≥ 12 years)	5	1.2
**Number of ASM**	Not on medication	2	0.5
	Monotherapy	320	74.9
	Polytherapy	105	24.6
**Neurodevelopmental comorbidity**	No neurodevelopmental comorbidity	297	69.6
	≥ 1 neurodevelopmental comorbidity	130	30.4
**Frequency of seizures in the past one month**	No episode	286	67.0
	1-2 episodes	106	24.8
	≥ 3 episodes	35	8.2
**Epilepsy related injuries**	No injury	385	90.2
	Injury	42	9.8
**Number of hospital admissions**	No admission	172	40.3
	1-2 admissions	209	48.9
	≥ 3 admissions	46	10.8

***Foot notes: SD = standard deviation; ASM = antiseizure medication.***

A statistically significant difference in seizure control was observed between epilepsy types (p = 0.003). Seizure freedom in the past month was higher among children with focal epilepsy (73.7%) compared to those with generalized epilepsy (59.5%). Conversely, poor seizure control (≥3 episodes) was more frequent in the generalized epilepsy group (11.8%) than in the focal epilepsy group (4.9%).

No other significant differences were found between the groups regarding age, sex, education level, age at seizure onset, prevalence of neurodevelopmental comorbidities, history of epilepsy-related injuries, or frequency of hospital admissions (all p > 0.05). The full comparison of characteristics by epilepsy type is presented in Table 1.

Treatment patterns differed significantly by epilepsy type. Monotherapy was the predominant approach for both groups, used in 76.8% of children with focal and 73.6% of those with generalized epilepsy, with no significant difference in the proportion on polytherapy (p = 0.448).

Carbamazepine was prescribed to 94.6% of children with focal epilepsy compared to only 16.6% of those with generalized epilepsy (p < 0.001). Conversely, valproate was used in 96.4% of children with generalized epilepsy versus 15.6% of those with focal epilepsy (p < 0.001). The use of other medications—phenobarbitone, levetiracetam, and clonazepam—did not differ significantly between the two groups (all p > 0.05). The full comparison of the treatment patterns is provided in Table 2.

To identify factors independently associated with seizure control, we performed a multivariate ordinal logistic regression. The multivariable ordinal logistic regression analysis is presented in Table 3. In the model, generalized epilepsy was independently associated with worse seizure control. Children with generalized epilepsy had 1.93 times higher odds (aOR=1.93, 95% CI: 1.28–2.92; p = 0.002) of being in a more severe seizure control category compared to those with focal epilepsy.

In contrast, age at seizure onset, treatment with polytherapy (versus monotherapy), and the presence of neurodevelopmental comorbidities were not significantly associated with seizure control in this model (all p > 0.05).

## Discussion

In this study of children with epilepsy at a tertiary hospital in Tanzania, we observed a slight male predominance (55%), a finding similar to reports in other settings in Sub-Saharan Africa (SSA), including a study in Uganda and with global epidemiological data [7,8]. The male disparity has been postulated in other research to be due to greater susceptibility to adverse perinatal outcomes in males, such as hypoxic ischemic encephalopathy (HIE) and other neurodevelopmental disorders, which may increase the risk of epilepsy [9].

Focal epilepsy was the predominant epilepsy type, affecting just over half of the children (52.5%). This is in keeping with a study from the United States of America (USA) where 68% of new-onset childhood epilepsy cases have a focal onset [10]. Similar findings have been reported in a narrative review by Camfield et al [3], and population-based studies in high-income countries (HIC) [11,12].

However, as this study was conducted in a tertiary referral center, the observed predominance of focal epilepsy may reflect referral patterns and the spectrum of epilepsy cases seen at tertiary-level care. It may not fully represent the distribution of epilepsy types in the general population.

Notwithstanding these setting-related considerations, the predominance of focal epilepsy in children is partly explained by its association with structural and metabolic etiologies, which are common early in life. In a population-based US cohort, structural and metabolic causes were the leading identifiable causes of childhood epilepsy and were predominantly observed in focal-onset epilepsy. In contrast, generalized epilepsies constituted a smaller proportion of the cohort and were primarily of genetic etiology (79%) [10]. This difference in etiological distribution may partly explain the higher prevalence of focal epilepsy in pediatric populations.

In our study, more than half (65.8%) of the children had an infantile onset of seizures, with the frequency decreasing with age. This is in keeping with population-based cohort studies and epidemiological reviews, which show the peak incidence in the first year of life and a subsequent decline [10,13]. This early onset is attributed to a higher burden of structural etiologies early in life, such as congenital brain malformations, HIE, perinatal and infantile Central nervous system (CNS) infections, which disrupt the developing brain and predispose to early-onset epilepsy [10].

Notably, we also found that a majority of children were on monotherapy (75.3%). This is in tandem with international epilepsy management guidelines that recommend starting with a single ASM to minimize side effects, improve adherence, and simplify the regimen [14].

The high rate of monotherapy in our cohort—despite being drawn from a tertiary referral center— may reflect effective first-line treatment for many patients, as evidenced by the two-thirds (67.0%) who were seizure-free in the month before the study. This is in keeping with existing evidence that suggests approximately half of epilepsy patients achieve seizure freedom with their first prescribed ASM [4]. Nevertheless, the considerable proportion of polytherapy (24.6%) underscores that a significant subset of children required combination therapy, likely reflecting more difficult-to-treat or refractory epilepsy. These patterns underscore the importance of ensuring consistent access to both first- and second-line ASM in resource-limited settings like Tanzania.

Regarding specific medication choices, our findings demonstrate a strong and guideline-concordant differentiation in treatment patterns by epilepsy type (Table 2). Valproate was predominantly prescribed for generalized epilepsy, used in 96.4% of those children compared to only 15.6% in the focal epilepsy group (p < 0.001). Conversely, carbamazepine was the mainstay for focal epilepsy, prescribed to 94.6% of those children versus 16.6% in the generalized group (p < 0.001). This treatment pattern aligns with international guidelines where narrow-spectrum agents such as carbamazepine are preferred for focal epilepsy [1], while broad-spectrum drugs, including valproate, are recommended as first line for generalized epilepsy due to their higher efficacy compared to other alternatives [6]. Notably, the use of other ASMs—including phenobarbitone, levetiracetam, and clonazepam—did not differ significantly between the two groups. Newer agents, particularly levetiracetam, were infrequently used, accounting for 1.9% of children receiving ASMs.

In our multivariate analysis, generalized epilepsy was independently associated with worse seizure control compared with focal epilepsy. This aligns with prospective studies showing that generalized epilepsy syndromes of unknown or structural etiology — historically termed “cryptogenic” or “symptomatic” — carry a higher risk of early intractability [15]. While these studies emphasize syndromic and etiologic classification rather than seizure type alone, our results suggest that, within this tertiary-care cohort in LMICs, children with generalized epilepsy may represent a subgroup with a higher likelihood of poor seizure control.

The lack of an observed association between polytherapy and seizure control should be interpreted in the context of routine clinical practice, where polytherapy is typically prescribed to children with more severe or drug-resistant epilepsy. As baseline epilepsy severity was not fully captured in this secondary analysis, confounding by indication may have limited the ability to detect an independent association between treatment regimen and seizure control.

Similarly, the lack of an observed association between neurodevelopmental comorbidities and seizure control should be interpreted cautiously. In this analysis, neurodevelopmental comorbidity was treated as a binary variable indicating the presence of at least one condition, without accounting for the type or severity of the conditions. This approach, while appropriate given sample size considerations, may have obscured differential effects of specific comorbidities or levels of impairment; additionally, limited statistical power to examine individual conditions separately may have contributed to the non-significant finding.

By drawing on a well-characterized cohort of 427 children with epilepsy at a national referral hospital, this study provides valuable real-world data on pediatric epilepsy care in LMIC settings, where such evidence remains limited. Epilepsy classification and ASM data were based on pediatric neurologist documentation and medical record review. Epilepsy types were classified in accordance with the International League Against Epilepsy (ILAE) 2017 operational classification, strengthening the validity and comparability of the findings.

Furthermore, seizure control was analyzed using ordinal logistic regression, which allowed seizure outcomes to be modeled across three clinically meaningful ordered categories (seizure-free, well-controlled, poorly controlled). This preserved important information on disease severity that would have been lost through dichotomization.

Our study has several limitations. First, the cross-sectional design and secondary analysis limit the establishment of causal relationships between epilepsy type, treatment patterns, and seizure control. Second, because the data were derived from a primary study that required caregivers to have provided care for at least six months before enrollment, the study population may preferentially represent children with more established or chronic epilepsy, potentially underrepresenting those with newly diagnosed epilepsy or severe forms of epilepsy. Moreover, as the study was conducted at a tertiary referral center, the findings may be more reflective of epilepsy patterns among children receiving ongoing specialist care, which may limit generalizability beyond similar settings. Third, although epilepsy diagnoses were made by a pediatric neurologist using detailed clinical semiology and supported by electroencephalography and neuroimaging, where clinically indicated during routine care, these investigations were not systematically captured as analyzable variables for the primary study and were therefore unavailable for inclusion in this secondary analysis. Although the classification reflects routine specialist clinical practice, the absence of uniformly collected EEG data in the analytic dataset may have introduced some potential for misclassification between focal and generalized epilepsy. This limitation should be considered when interpreting findings related to epilepsy type. Finally, seizure control was assessed using caregiver-reported seizure frequency over the preceding month. Although this shorter recall period was chosen to improve accuracy in a clinic-based setting, it may not fully capture longer-term seizure patterns. While seizure diaries were used to verify seizure frequency when available, not all caregivers presented with diaries; therefore, reliance on caregiver recall for some participants may have introduced under- or over-reporting and should be considered when interpreting the findings.

## Conclusion

This study found that focal epilepsy is the predominant type among children attending a tertiary referral hospital in Tanzania, with treatment patterns largely aligned with international guidelines. Children with generalized epilepsy had poorer seizure control in comparison to those with focal epilepsy. Further prospective studies incorporating detailed syndromic and etiologic classification are needed to better define predictors of seizure outcomes and to inform service planning.

## Supporting information

S1 File Published study report: Depression, anxiety, and associated factors among caregivers of children and adolescents with epilepsy attending Muhimbili Pediatric Neurology Clinic: a cross-sectional study. (PDF)

S2 File Checklist for Reporting of Survey Studies (CROSS). (PDF)
