# Supplementary material for: Clinical characteristics, treatment patterns, and seizure control among children with focal and generalized epilepsy at a tertiary hospital in Tanzania: A cross-sectional secondary analysis
Source: PLoS One. 2026 Apr 2;21(4):e0344724. doi: 10.1371/journal.pone.0344724 (PMC13046136; doi:10.1371/journal.pone.0344724)
Supplement: S2 File — (PDF) [file pone.0344724.s002.pdf]

SF_2 Checklist for Reporting Of Survey Studies (CROSS)

Section/topic	Item	Item description	Reported on page #
**Title and abstract**			
Title and abstract	1a	State the word "survey" along with a commonly used term in title or abstract to introduce the study's design.	**Title:** Cross-sectional secondary analysis
	1b	Provide an informative summary in the abstract, covering background, objectives, methods, findings/results, interpretation/discussion, and conclusions.	• **Abstract: Page 1–2**
**Introduction**			
Background	2	Provide a background about the rationale of study, what has been previously done, and why this survey is needed.	• **Background: Introduction (Page 2–4)**
Purpose/aim	3	Identify specific purposes, aims, goals, or objectives of the study.	• **End of Introduction (Page 4)**
**Methods**			
Study design	4	Specify the study design in the methods section with a commonly used term (e.g., cross-sectional or longitudinal).	**Materials and methods (Page 4–5)**
	5a	Describe the questionnaire (e.g., number of sections, number of questions, number and names of instruments used).	**Materials and methods Page 4**<br>Not applicable – secondary analysis of existing dataset
Data collection methods	5b	Describe all questionnaire instruments that were used in the survey to measure particular concepts. Report target population, reported validity and reliability information, scoring/classification procedure, and reference links (if any).	**Data collection**<br>Page 5-6
	5c	Provide information on pretesting of the questionnaire, if performed (in the article or in an online supplement). Report the method of pretesting, number of times questionnaire was pre-tested, number and demographics of participants used for pretesting, and the level of similarity of demographics between pre-testing participants and sample population.	Not applicable
	5d	Questionnaire if possible, should be fully provided (in the article, or as appendices or as an online supplement).	Not applicable

Sample characteristics	6a	Describe the study population (i.e., background, locations, eligibility criteria for participant inclusion in survey, exclusion criteria).	**Materials and methods** Page 5<br>Study population
	6b	Describe the sampling techniques used (e.g., single stage or multistage sampling, simple random sampling, stratified sampling, cluster sampling, convenience sampling). Specify the locations of sample participants whenever clustered sampling was applied.	**Materials and methods Page 5**<br>Consecutive enrolment in primary study
	6c	Provide information on sample size, along with details of sample size calculation.	**Materials and methods Page 5**<br>Sample size described (n=427)
	6d	Describe how representative the sample is of the study population (or target population if possible), particularly for population-based surveys.	**Results Page 9-12**<br>Tables 1–2
Survey administration	7a	Provide information on modes of questionnaire administration, including the type and number of contacts, the location where the survey was conducted (e.g., outpatient room or by use of online tools, such as SurveyMonkey).	**Data collection Page 5-6**<br>Interviews + medical records
	7b	Provide information of survey's time frame, such as periods of recruitment, exposure, and follow-up days.	**Data collection Page 5**<br>June–October 2023
	7c	Provide information on the entry process:<br>–>For non-web-based surveys, provide approaches to minimize human error in data entry.<br>–>For web-based surveys, provide approaches to prevent "multiple participation" of participants.	**Materials and methods**<br>**Page 7-8**
Study preparation	8	Describe any preparation process before conducting the survey (e.g., interviewers' training process, advertising the survey).	**Materials and methods Page 5-6**<br>Training of research assistant described
Ethical considerations	9a	Provide information on ethical approval for the survey if obtained, including informed consent, institutional review board [IRB] approval, Helsinki declaration, and good clinical practice [GCP] declaration (as appropriate).	**Ethical Statement Page 7**<br>Approval primary study (MUHAS-REC-05-2023-1679)
	9b	Provide information about survey anonymity and confidentiality and describe what mechanisms were used to protect unauthorized access.	**Ethical Statement Page 7**

			Confidentiality and data protection described
Statistical analysis	10a	Describe statistical methods and analytical approach. Report the statistical software that was used for data analysis.	**Data analysis Page 7**<br>STATA 18
	10b	Report any modification of variables used in the analysis, along with reference (if available).	**Data analysis Page 8**<br>• Outcome categorization: Data analysis – seizure control categorized into three ordered levels
	10c	Report details about how missing data was handled. Include rate of missing items, missing data mechanism (i.e., missing completely at random [MCAR], missing at random [MAR] or missing not at random [MNAR]) and methods used to deal with missing data (e.g., multiple imputation).	**Results Page 9**<br>No missing data reported
	10d	State how non-response error was addressed.	**Materials and methods Page 5**<br>All eligible participants included)
	10e	For longitudinal surveys, state how loss to follow-up was addressed.	N/A
	10f	Indicate whether any methods such as weighting of items or propensity scores have been used to adjust for non-representativeness of the sample.	N/A
	10g	Describe any sensitivity analysis conducted.	N/A
**Results**			
Respondent characteristics	11a	Report numbers of individuals at each stage of the study. Consider using a flow diagram, if possible.	**Results Page 9**<br>n=427 enrolled
	11b	Provide reasons for non-participation at each stage, if possible.	**Materials and methods Page 5**<br>Reasons for exclusion in primary described
	11c	Report response rate, present the definition of response rate or the formula used to calculate response rate.	Response rate reported (100%)

	11d	Provide information to define how unique visitors are determined. Report number of unique visitors along with relevant proportions (e.g., view proportion, participation proportion, completion proportion).	**N/A**
Descriptive results	12	Provide characteristics of study participants, as well as information on potential confounders and assessed outcomes.	**Results Page 9–12**<br>Table 1-2
Main findings	13a	Give unadjusted estimates and, if applicable, confounder-adjusted estimates along with 95% confidence intervals and p-values.	Results **Page 13-14**<br>Table 3-4
	13b	For multivariable analysis, provide information on the model building process, model fit statistics, and model assumptions (as appropriate).	**Data analysis Page 8**<br>Model building and assumptions described
	13c	Provide details about any sensitivity analysis performed. If there are considerable amount of missing data, report sensitivity analyses comparing the results of complete cases with that of the imputed dataset (if possible).	N/A
**Discussion**			
Limitations	14	Discuss the limitations of the study, considering sources of potential biases and imprecisions, such as non-representativeness of sample, study design, important uncontrolled confounders.	**Discussion – Limitations subsection Page 18–19**
Interpretations	15	Give a cautious overall interpretation of results, based on potential biases and imprecisions and suggest areas for future research.	**Discussion Page 15-19**
Generalizability	16	Discuss the external validity of the results.	**Discussion Page 18**<br>Addressed in the tertiary referral center paragraph
**Other sections**			
Role of funding source	17	State whether any funding organization has had any roles in the survey's design, implementation, and analysis.	• **Funding: Funding section Page 20**
Conflict of interest	18	Declare any potential conflict of interest.	• **Competing interests: Competing interests section Page 20**
Acknowledgements	19	Provide names of organizations/persons that are acknowledged along with their contribution to the research.	• Acknowledgments section **Page 19-20**
